# Giant cell carcinoma of the lung with hypertrophic pulmonary osteoarthropathy as a paraneoplastic syndrome: a case report

**DOI:** 10.1186/s44215-023-00067-w

**Published:** 2023-08-17

**Authors:** Akimu Sobue, Teruaki Mizobuchi, Fumihiro Ishibashi, Kaoru Nagato, Hiroki Imabayashi, Yasuo Ishida, Isamu Sugano

**Affiliations:** 1 Department of General Thoracic Surgery, Social Welfare Organization Saiseikai Imperial Gift Foundation, Chibaken Saiseikai Narashino Hospital, 1-8-1 Izumi-Cho, Narashino-Shi, Chiba-Ken 275-8580 Japan; 2 Department of Pathology, Social Welfare Organization Saiseikai Imperial Gift Foundation, Chibaken Saiseikai Narashino Hospital, Narashino-Shi, Chiba Japan

**Keywords:** Paraneoplastic syndrome, Hypertrophic pulmonary osteoarthropathy, Giant cell carcinoma of the lung

## Abstract

**Background:**

Hypertrophic pulmonary osteoarthropathy is a rare syndrome characterized by a triad that includes periostitis, digital clubbing, and painful arthropathy of the large joints, especially large joints in the lower limbs with lung cancer. Herein we describe a case of a giant cell carcinoma of the lung with hypertrophic pulmonary osteoarthropathy as a paraneoplastic syndrome. The tumor was successfully resected, and complete remission of the syndrome was achieved after surgery.

**Case presentation:**

A 48-year-old man with right fingers and bilateral ankle arthralgias was referred to our hospital. These arthropathies were painful and refractory to the oral administration of several non-steroidal anti-inflammatory drugs; thus, oxycodone hydrochloride hydrate was prescribed. Additionally, the fingers and toes had a clubbed appearance and the tubular bones were shown as double lines on radiographs of both arms and legs, indicating periosteal thickening. A 45-mm mass-like shadow was present on the right upper-to-middle lung field on the chest x-ray, which was confirmed to be a mass in the upper lobe of the right lung on the chest CT scan. An abnormal uptake was observed in the area of the mass on the FDG-PET scan; the standardized uptake value maximum was 11.8. The histologic diagnosis of a bronchoscopic biopsy was an undifferentiated carcinoma, and the clinical diagnosis was non-small cell lung cancer. The clinical stage was c-T2bN0M0 with paraneoplastic syndrome as a hypertrophic pulmonary osteoarthropathy. A right upper lobectomy with an S6 partial resection adjacent to the upper lobe of the right lung and an ND2a-2 lymph node dissection was performed. The postoperative course was uneventful. The pathologic diagnosis was a giant cell lung carcinoma, which was classified as p-T3 (52 mm in diameter) N0M0, stageIIB. A gradual decrease in arthritic pain was observed from the first operative day. No anodyne, including oxycodone, was required approximately 2 months after surgery. No tumor or paraneoplastic syndrome recurrence was observed 2 years postoperatively.

**Conclusions:**

Hypertrophic pulmonary osteoarthritis may occur as a secondary manifestation of lung malignancies. Thus, the paraneoplastic syndrome should be considered in such cases.

## Background

Hypertrophic pulmonary osteoarthropathy (HPOA) is a paraneoplastic syndrome that occurs in patients with lung cancer. The triad of clinical signs includes finger clubbing, polyarthritis, and periostitis of the tubular bones [1]. Giant cell cancer of the lung (GCCL) has been reported as a relatively rare malignant tumor with a poor prognosis. We report herein an uncommon combination of a GCCL and HPOA, which were successfully treated by lung resection.

## Case presentation

**Fig. 1:**
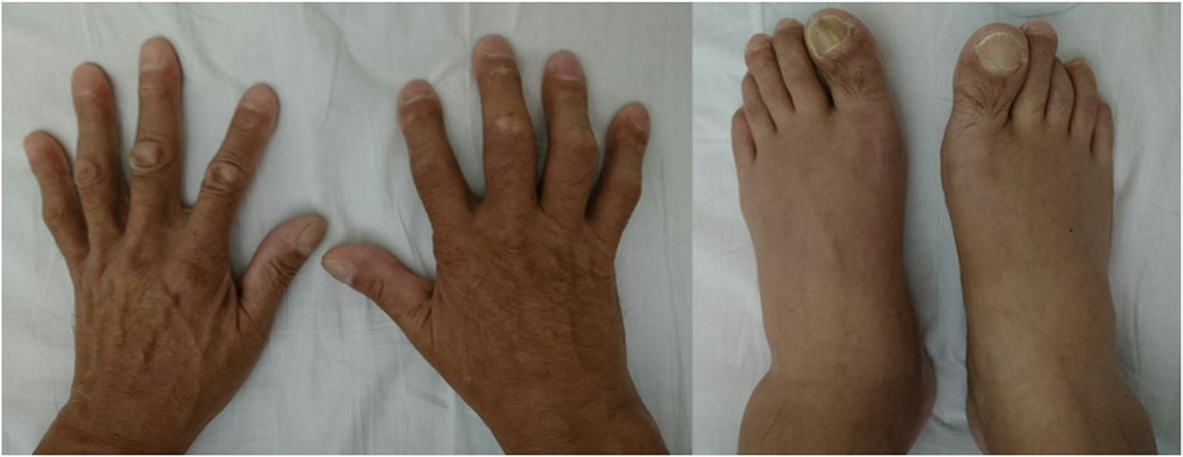
Clubbing of the fingers and swelling of the legs were observed on the physical exam

**Fig. 2:**
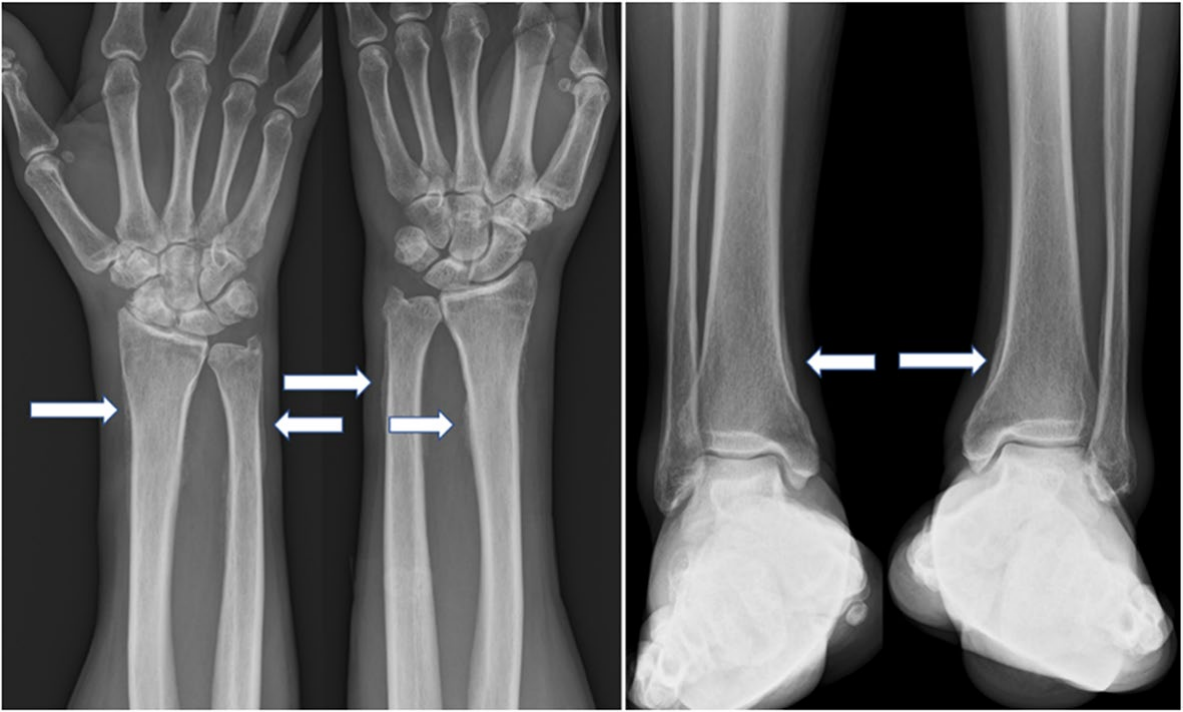
X-rays showed double lines on the radiuses, tibias, and fibulas, consistent with periosteal thickening

**Fig. 3:**
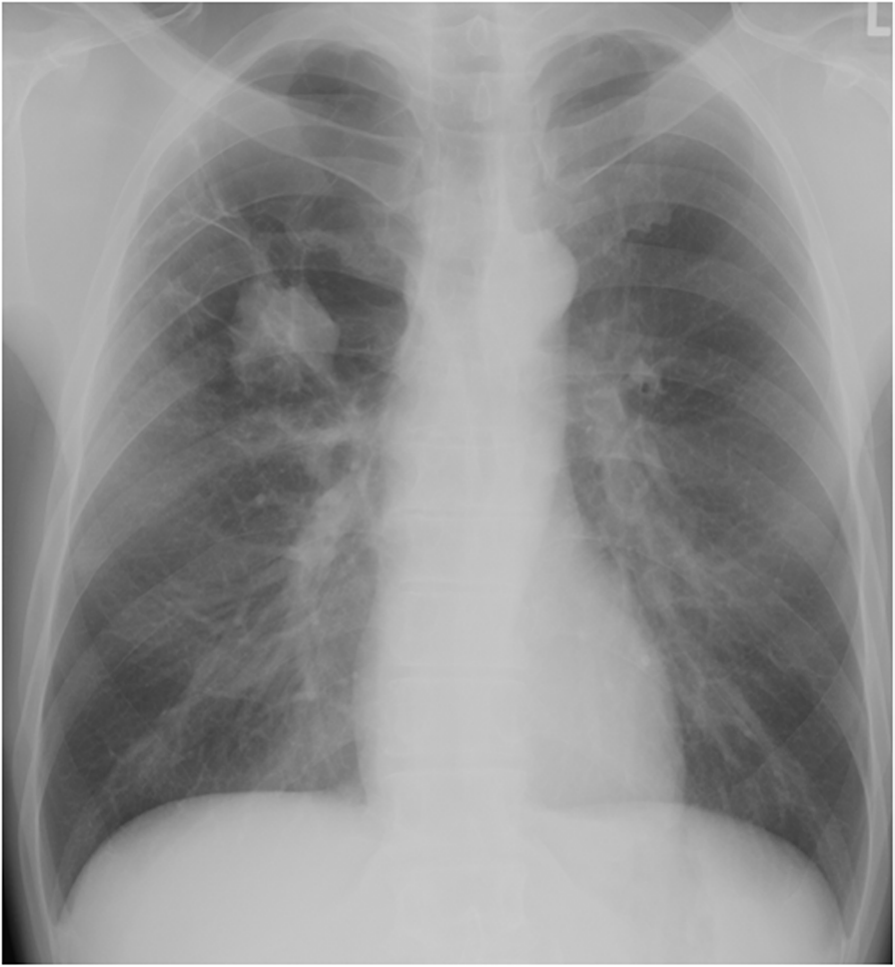
Chest X-ray showed a 44-mm mass in the right middle lung area

**Fig. 4:**
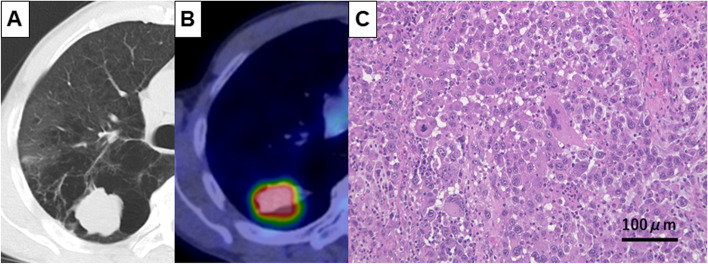
A chest CT showed a 45-mm solid mass with indistinctive borders and irregular edges in the right upper lobe (**A**), which had a high 18F-fluorodeoxyglucose uptake (SUVmax = 11.8) (**B**). After surgery, the pathologic examination showed a giant cell carcinoma in which mono- or poly-nuclear tumor cells with large polygonal eosinophilic endoplasmic reticula and prominent nuclear bodies were seen in a dense arrangement (**C**)

**Fig. 5:**
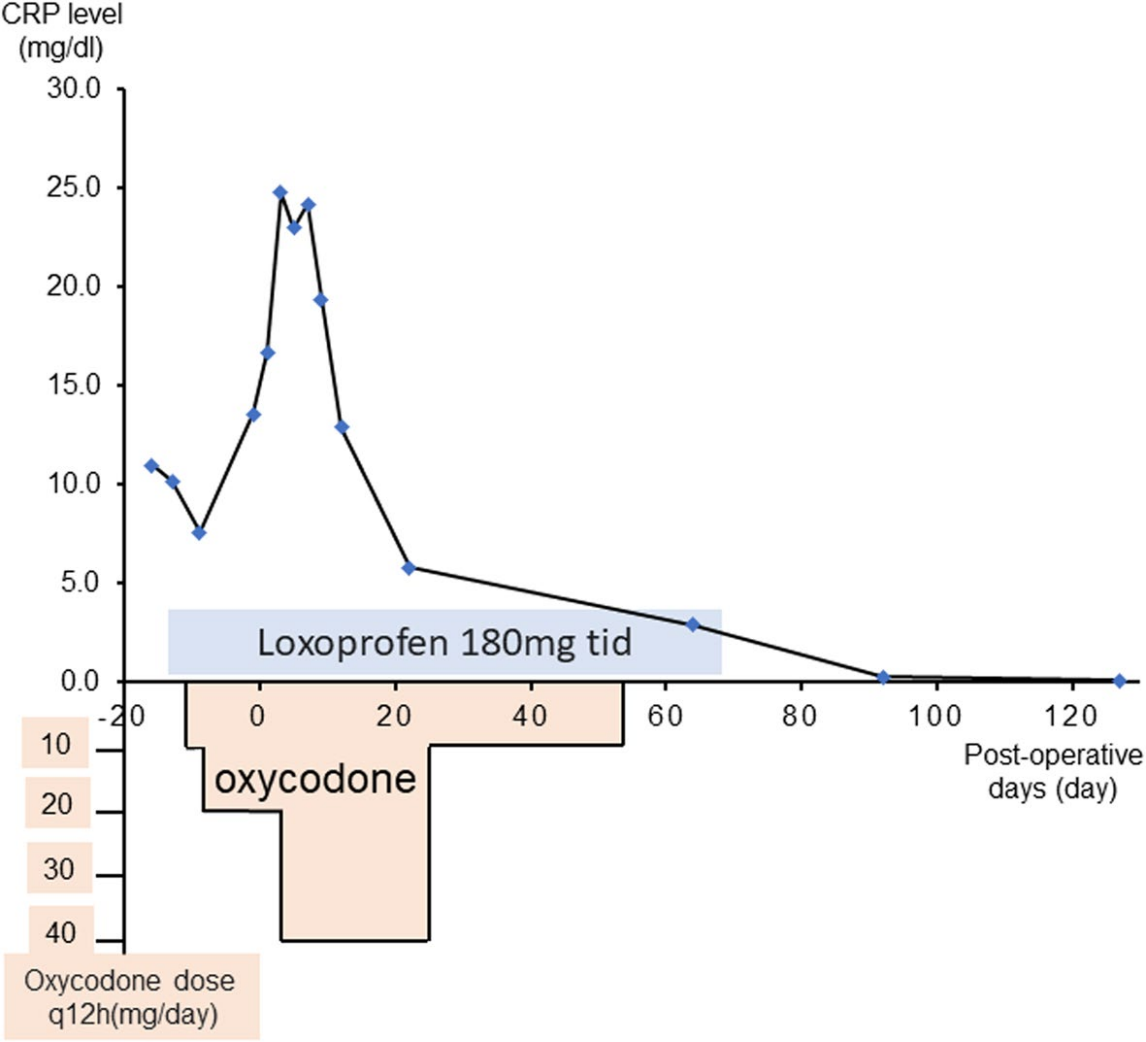
Changes in CRP Level and pain medication content and dosage before and after surgery

A 48-year-old man with complaints of swelling and pain in the bilateral foot and right finger joints was referred to an orthopedic clinic. The oral medications that were prescribed, including non-steroidal anti-inflammatory drugs and colchicine, did not ameliorate the symptoms; thus, he was referred to our hospital for further evaluation and treatment. The physical examination showed clubbing of the fingers on both hands and swelling of the legs (Fig. 1). X-rays revealed double lines bilaterally on the distal aspects of the radius and ulna, and distal tibiae, suggesting periosteal thickening (Fig. 2). Suspecting collagen disease-related arthritis, the patient was referred to the department of collagen disease at first. Based on negative rheumatoid factor, negative antinuclear antibody, negative ferritin level, and low uric acid level, he was suspected to have arthritis related to calcium pyrophosphate dihydrate deposition, or paraneoplastic syndrome as a differential diagnosis. Accordingly, the patient was screened for malignant disease and a mass suspected to be lung cancer was detected, which led to a referral to our general thoracic surgery department. The incidental mass shadow was noted in the right upper-middle lung field on the chest X-ray (Fig. 3). A chest CT scan confirmed a 45-mm solid mass with an irregular edge and indistinctive border in the right upper lobe (Fig. 4A), which had high 18F-fluorodeoxyglucose uptake (standardized uptake value maximum [SUVmax] = 11.8; Fig. 4B). Blood testing revealed mild anemia with severe inflammation. The c-reactive protein (CRP) level was high at 13.6 mg/dl before the surgery. Tumor marker levels, including CEA, CYFRA, and ProGRP, were all within normal limits. A cytologic examination of a transbronchial aspiration from the mass showed undifferentiated carcinoma cells. The clinical diagnosis was a c-T2bN0M0 non-small cell lung cancer and paraneoplastic syndrome with swelling of the distal limb bones and joints, periosteal overlapping signs, and clubbing of the fingers, which was diagnosed as an HPOA. Pain control was incomplete despite the use of 180 mg of loxoprofen sodium per day, three times a day; therefore, an additional 40 mg of oxycodone hydrochloride hydrate daily with rescue doses was necessary for pain control preoperatively (Fig. 5). Under general anesthesia with one-lung ventilation, an upper lobectomy and S6 partial resection of the right lung with an ND2a-2 lymph node dissection were performed. The pathologic examination showed a 52-mm giant cell carcinoma composed of mono- or poly-nuclear tumor cells with large polygonal eosinophilic endoplasmic reticula and prominent nuclear bodies (Fig. 4C). No invasion of the tumor into the S6 segment was demonstrated; accordingly, the pathologic stage was pT3N0M0. The HPOA pain had gradually decreased by postoperative day 1, and he was pain-free without any analgesic 64 days later and the high CRP level decreased within the normal limit 3 months after the tumor removal according to an improvement of the HPOA symptoms (Fig. 5). No tumor recurrence was detected as of the 2-year follow-up evaluation.

## Discussion and conclusions

HPOA is a paraneoplastic syndrome that is characterized by a triad consisting of finger clubbing, polyarthritis, and tubular bone periostitis with lung cancer. In addition to the clinical findings, bone scintigraphy is useful to establish the diagnosis of HPOA [1]. HPOA occurs in 0.2–0.8% of patients with lung cancer, concerning the underlying malignancy, adenocarcinoma, and squamous cell carcinoma account for the majority of HPOA cases; on the other hand, small cell carcinoma involvement was reported to be extremely rare [1–3]. Several hypotheses about the etiology of HPOA have been proposed, such as growth hormone excess and VEGF elevation [4]. Histologically, HPOA begins as an inflammatory reaction with periosteal edema, cell infiltration, and angiogenesis. Then, there is a shift to chronic proliferative changes, which manifest as a periosteal thickening or bone neo-plasticity [5]. Namely, HPOA is a rare inflammatory syndrome affecting bones and joints, but it is considered to be caused by substances released from the tumor or by the body’s response to the tumor. Therefore, after complete resection of the tumor, the inflammatory signs related to HPOA improve spontaneously and the administration of anti-inflammatory and analgesic drugs is no longer necessary. In our patient with GCCL, the postoperative necessity of non-steroidal anti-inflammatory drugs and oxycodone was eliminated, and the C-reactive protein levels normalized, which would reversely prove that the arthritis was related to the paraneoplastic syndrome with GCCL.

GCCL was first reported by Nash and Stout in 1958 [6]. According to the 2015 WHO classification, pleomorphic carcinoma, spindle cell carcinoma, and GCCL are described as one category. In this section, GCCL is defined to consist almost entirely of tumor giant cells with no differentiated carcinomatous elements [7]. While GCCL is classified as a sarcomatoid carcinoma, sarcomatoid carcinoma is an unusual form of non-small cell lung cancer that comprises 0.1 to 0.4% of all pulmonary malignancies [8, 9]. Accordingly, pure GCCL is very rare [9]. The definitive diagnosis of GCCL relies on a histopathologic evaluation of the resected tumor instead of cytology and small biopsy specimens.

GCCL is more likely to metastasize and progress more rapidly than other NSCLC subtypes. Surgical resection is the primary option for GCCL treatment; however, many patients are not surgical candidates because the tumor has metastasized at the time of diagnosis [10]. Platinum-based chemotherapy has been commonly used to treat GCCL, but numerous studies have demonstrated a poor response to chemotherapy [11]. Recently, tumor-related driver genes have been investigated for advanced GCCL. Several researchers report that GCCL patients with sensitive *EGFR* mutations may also benefit from *EGFR*-TKI [10], or treatment using MEK inhibitor, CDK 4/6 inhibitor, and TP53 inhibitor may provide a new therapeutic direction for GCCL [11]. Accordingly, complete tumor excision is the best choice of treatment strategy at the early stage of GCCL; on the other hand, these gene target therapies may become a pivotal option for this disease in the future.

Paraneoplastic syndrome co-exists with GCCL, as evidenced by a case of gynecomastia attributed to a human chorionic gonadotropin-secreting GCCL [12] and a case of hypertrophic osteoarthropathy with GCCL [13]. The paraneoplastic syndrome in the latter case was an HPOA that improved after surgical resection of the GCCL. In our case, conventional tumor markers for lung cancer were negative; on the other hand, the c-reactive protein (CRP) level was high at 13.6 mg/dl before the surgery. The high CRP level decreased within the normal limit after the tumor removal according to an improvement of the HPOA symptoms. The presence of HPOA as well as the presence of cancer generates inflammation, leading to an increase in CRP [14]. Additionally, Yuan and colleagues reported on primary hypertrophic osteoarthropathy (PHO), a similar disease to HPOA, that a cyclooxygenase-2 inhibitor improved PHO symptoms in a majority of the patients and that high CRP levels before treatment were significantly improved after treatment [15]. Hence, CRP may be one of the indicators in the follow-up of HPOA.

In conclusion, we have reported a rare paraneoplastic syndrome with HPOA attributed to GCCL that was successfully treated by conventional lung cancer surgery.

## Data Availability

The data of this manuscript are all included in the case report.

## Abbreviations

HPOA: Hypertrophic pulmonary osteoarthropathy
GCCL: Giant cell cancer of the lung
